# Association of dietary pattern and physical activity with lipid-related indices among Chinese population: a cross-sectional study

**DOI:** 10.1186/s12944-020-01420-6

**Published:** 2020-11-23

**Authors:** Qiao Guo, Zuchang Ma, Changan Zhu, Qiang Zeng

**Affiliations:** 1 Precision Machinery and Precision Instruments, Institute of Engineering and Science, University of Science and Technology of China, Hefei, 230026 Anhui People’s Republic of China; 2 Institute of Intelligent Machines, Hefei Institutes of Physical Science, Chinese Academy of Sciences, Hefei, 230031 Anhui People’s Republic of China; 3 Institute of Health Management, Chinese People’s Liberation Army (PLA) General Hospital, Beijing, 100853 People’s Republic of China; 4 Hefei, China

**Keywords:** Dietary pattern, Physical activity level, Lipid-related indices, Chinese population, Cross-sectional study

## Abstract

**Background and objectives:**

To explore the relationship between dietary patterns, physical activity and lipid-related indices in Chinese Population.

**Methods and study design:**

This study included 21,472 (72.3% men) participants aged 16 to 78 years. Data of anthropometric measurements, biochemical tests and questionnaires were collected through a physical examination. Diet patterns were identified through factor analysis and five patterns were retained (“meat,” “high-energy,” “high-protein,” “healthy” and “traditional Chinese”). Physical activity was classified into low, moderate, or high. Abnormalities in lipid indices were assessed using the Adult Treatment Panel III criterion.

**Results:**

Higher factor scores of “high-protein” pattern and “healthy” pattern were found to be related to favorable lipid indices. Quartiles 3 and 4 of “meat” pattern showed increased risks of having elevates total cholesterol and low-density lipoprotein cholesterol concentrations. Participants with higher levels of physical activity showed lowest risk of abnormal lipid profiles. All the associations were equally established among men, while most were no longer significant among women.

**Conclusions:**

Higher physical activity level and a dietary pattern consists of high-quality protein foods, vegetables and fruits were associated with favorable lipid profiles, and these lifestyle factors were related to the risk of dyslipidemia in a sex-specific way.

## Introduction

Dyslipidemia has become an important public health problem worldwide due to its risk of development of cardiovascular diseases and high prevalence [1–3]. Prevalence of dyslipidemia in China has increased rapidly in past decades [4], and cardiovascular diseases and events caused by dyslipidemia is expected to increase by 9.2 million between 2010 and 2030 [5]. Diet and PA are the two important controllable lifestyle factors in management of various chronic disease, the potential influence on lipid profiles of these factors are considerable [6, 7].

Good lifestyle habits including healthy eating habits and regular exercise have been known to be beneficial to various chronic conditions and diseases including dyslipidemia [8–15]. The Dietary Approaches to Stop Hypertension which recommended a diet to consume more fruits and vegetables and limited in saturated fats and cholesterol products has been proven to be beneficial to low-density lipoprotein cholesterol (LDL-C) level [8, 9]. Some other studies have revealed that diet consumes more saturated fat and sugar is associated with dyslipidemia [10, 11]. Physical activity (PA) has been proven to be beneficial to lipid profiles by elevate high-density lipoprotein cholesterol (HDL-C) and lowering triglyceride (TG) levels [12–14], and its mechanism may be due to the improvement of endothelial function [15].

Other studies suggested that dietary factors are also related to lipids and the risk of developing chronic non-communicable diseases. For example, high proportion of saturated fatty acids, refined carbohydrates, and less intake of n-3 polyunsaturated fatty acids (n-3 PUFA) were found to be related to the increased risk of non-alcoholic fatty liver disease (NAFLD), which is related to circumstances including hyperlipidemia and obesity [16, 17]. However, due to China’s large geographical span, complex and diverse eating habits are found across regions, and information on the association between dietary pattern, physical activity level (PAL) and lipid profiles in the Chinese population must be supplemented.

Diet and PAL are important elements in the management of dyslipidemia, because better dietary pattern and increased PA may lead to better health outcomes. However, the optimal dietary pattern for Chinese population and its interaction with PA remain unclear. A cross-sectional analysis was performed on the Chinese population to examine the associations between dietary pattern and PAL with lipid. Dietary pattern and PAL were selected as influencing factors and the odds ratios (ORs) of each pattern and PAL were used to evaluate the risk of abnormal lipid-related indices. Novel approaches for the management of lipid profile and improvement in the understanding of the associations between diet and PA with lipid-related indices are expected through the results of this study.

## Materials and methods

### Participants

Screening was conducted for 24,521 potential subjects aged 16 to 78 years who received a health examination from August 2012 to July 2015 at the physical examination center of Beijing, China were screened. After excluded 3049 subjects due to lack of data on diet habit or PA, 21472 (72.3% men) subjects were included. Informed consent was obtained from all subjects before the examination commenced. This study was conducted in accordance with ethical standards.

### Sociodemographic and anthropometric characteristics

Demographic characteristics, lifestyle characteristics, and personal and family medical history data were collected by an interview with trained and certified personnel using a standard questionnaire. A physical examination was conducted for each participant after the interview. Weight was measured to the nearest 0.1 kg. Height and waist circumstance (WC) were measured to the nearest 0.1 cm. Body mass index (BMI) was calculated as weight in kilograms divided by the square of the height in meters.

### Dietary patterns

Dietary composition data in the past three months were obtained through a retrospective diet frequency questionnaire. The reliability of this questionnaire has been verified [18]. A total of 16 types of food items, food groups and beverages were included in the questionnaire, which contained questions on frequency of consumption and average consumption of each food item, food group and beverage. Frequency was recorded by the number of times each food item was consumed per week, and the quantity consumed was recorded by “liang”, a local unit commonly used by Chinese people for weight (1 liang = 50 g), or cups (1 cup = 250 mL). The alcohol intake of the subjects in the past year was also obtained during the interview. Intake data were converted into averages in grams or cups per day for further analyses.

Factor analysis of food items and standard principal component analysis method were used to establish dietary patterns [19]. Data adequacy was assessed by the Kaiser–Meyer–Olkin and the Bartlett Test of Sphericity for factor analysis. The orthogonal rotation method was used on the factors to make them easier to explain and reduce the relevance between factors. In this study, the extraction threshold of factors is greater than or equal to 1, and individual food with an absolute factor score not less than 0.2 was considered to have a significant contribution to the pattern. Factor scores correspond to simple correlations between the food and the factor. The greater variance food shares with each factor was reflected by higher scores. The direction of each food to the factor is determined by the sign of factor score. Each factor was named descriptively based on its food structure.

Five major diet patterns were extracted: the “meat” pattern (high in meat, eggs, bean products, fish and alcohol); the “high-energy” pattern (high in sugary drinks, pickled food, fried food and sweets); the “high-protein” pattern (high in coarse cereals, eggs, dairy, bean products and soybean milk); the “healthy” pattern (high in grain, coarse cereals, vegetables and fruits while low in alcohol and salt); and the “traditional Chinese” pattern (high in grain, meat, pickled food, fried food, alcohol and salt while low in vegetables and fruits).

Factor scores of each diet pattern were categorized according to the quartiles for analysis. The scores increased form quartile 1 (Q1) to quartile 4 (Q4). In the logistic regression, categorical variables were treated as continuous variables for trend test. The main influencing variables and their product were included in logistic regression to evaluate the interaction. Dietary patterns were divided into tertiles of factor scores for the interaction calculation.

### PAL assessment

PA data were recorded as the frequency and time of walking, moderate and vigorous PA. PAL was classified into low, moderate, or high according International Physical Activity Questionnaire (IPAQ) scoring protocol [20]. Walking, moderate, and vigorous PA were considered as 3.3, 4.0, and 8.0 metabolic equivalents of tasks (METs), respectively. MET-minutes was obtained by multiplying the MET value of each intensity of PA by the total time per week of the corresponding PA and then accumulating the MET-minutes of each intensity.

### Biochemical measurements

Venous blood was obtained in the mornings after at least 10 h of fasting. Total cholesterol (TC), TG, HDL-C and LDL-C levels were collected. Serum TC, TG and HDL-C were analyzed using Hitachi 7150 auto-analyzer (Hitachi, Tokyo, Japan). LDL-C was calculated using the Friedewald formula: LDL-C = TC–HDL-C– (TG/2.2) [21]. The threshold used to identify TC, TG, and LDL-C abnormalities were 5.18, 1.70, and 3.37 mmol/L, respectively, and the threshold of low HDL-C is 1.04 mmol/L for men and 1.29 mmol/L for women, according to the definition of metabolic abnormalities by the Adult Treatment Panel III [22].

### Statistical analysis

Continuous variables were presented as mean ± standard deviation (SD), categorical variables were expressed as quantities and percentages. Odds ratios (ORs) with 95% confidence intervals (CIs) were calculated using logistic regression analysis. All statistical analyses were performed using commercially available software and significance was defined as *P* <  0.05.

## Results

The demographic information and ORs of blood lipid abnormalities according to sex are exhibited in Table 1. Higher BMI and excess WC were associated with increased risks of blood lipid abnormalities across genders. Alcohol drinking was observed to be associated with elevated risk of TC abnormalities and decreased risk of HDL-C abnormalities, while the relationship was different across genders in terms of the risk of TG abnormalities. In addition, smoking was found to be related to higher risk of TG abnormalities in men.

**Table 1:** Characteristics and ORs of abnormal lipid indices of the study participants according to categories of gender (*n* = 21,472)

	N (%)		OR (β)							
			High TC		High TG		Low HDL-C		High LDL-C	
	Men (*n* = 15,516)	Women (*n* = 5956)	Men	Women	Men	Women	Men	Women	Men	Women
Age (years)	47.7 ± 7.9	48.2 ± 8.3	–	–	–	–	–	–	–	–
Ethnicity^a^										
Han	14,822 (95.5)	5587 (93.8)	1	1	1	1	1	1	1	1
Other	694 (4.5)	369 (6.2)	0.95 (−0.06)	1.00 (0.00)	1.05 (0.05)	1.16 (0.15)	1.01 (0.01)	1.07 (0.07)	1.00 (0.00)	0.94 (−0.06)
Education^a^										
Universities	10,629 (68.5)	2976 (50.0)	1	1	1	1	1	1	1	1
High School	4523 (29.2)	2507 (42.1)	0.97 (0.08)	1.02 (0.02)	1.07 (0.07)	1.21 (0.19)’	1.28 (0.25)”	1.43 (0.36)”	0.96 (−0.04)	1.11 (− 0.10)
Primary school	364 (2.3)	473 (7.9)	1.09 (−0.03)	0.92 (−0.09)	0.81 (− 0.21)	1.06 (0.06)	1.12 (0.11)	1.38 (0.32)’	1.00 (0.00)	0.91 (0.10)
*P* for trend			0.79	0.69	0.54	0.08	< 0.001	< 0.01	0.35	0.73
BMI (kg/m^2^)^a^	25.7 ± 3.2	23.5 ± 3.3								
Low (≤18.5)	164 (1.1)	278 (4.7)	1	1	1	1	1	1	1	1
Normal (18.6–23.9)	4156 (26.8)	3311 (55.6)	2.63 (0.97)”	1.38 (0.32)	7.44 (2.01)”	6.47 (1.87)”	5.01 (1.61)”	4.68 (1.54)”	3.08 (1.13)”	2.69 (0.99)”
Overweight (24.0–27.9)	7858 (50.6)	1815 (30.5)	3.19 (1.16)”	1.49 (0.40)’	17.02 (2.84)”	13.29 (2.59)”	10.34 (2.34)”	10.50 (2.35)”	3.57 (1.27)”	3.47(1.24)”
Obesity (≥28.0)	3338 (21.5)	552 (9.3)	3.49 (1.25)”	1.39 (0.33)	23.43 (3.15)”	14.08 (2.65)”	14.46 (2.67)”	15.20 (2.72)”	3.79 (1.33)”	3.71(1.31)”
*P* for trend			< 0.001	0.15	< 0.001	< 0.001	< 0.001	< 0.001	< 0.001	< 0.001
WC (cm)^a^	91.6 ± 8.4	79.8 ± 8.5								
Normal (< 85 for men; < 80 for women)	2898 (18.7)	2998 (50.3)	1	1	1	1	1	1	1	1
Exceed (≥85 for men; ≥80 for women)	12,618 (81.3)	2958 (49.7)	1.51 (0.41)”	1.12 (0.12)	3.12 (1.14)”	2.63 (0.97)”	2.73 (1.01)”	2.77 (1.02)”	1.44 (0.37)”	1.37 (0.32)”
*P* for trend			< 0.001	0.06	< 0.001	< 0.001	< 0.001	< 0.001	< 0.001	< 0.001
Alcohol drinking^b^										
Never	2515 (16.2)	4316 (72.5)	1	1	1	1	1	1	1	1
Former	2035 (13.1)	665 (11.2)	0.94 (−0.06)	1.21 (0.19)’	1.10 (0.10)	1.09 (0.09)	1.10 (0.10)	1.01 (0.01)	0.93 (−0.07)	1.19 (0.17)
Current	10,966 (70.7)	975 (16.4)	1.48 (0.39)”	1.23 (0.21)’	1.66 (0.51)”	0.82 (−0.20)’	0.66 (− 0.42)”	0.73 (− 0.32)”	1.10 (0.09)	1.09 (0.09)
*P* for trend			< 0.001	< 0.05	< 0.001	0.11	< 0.001	< 0.05	< 0.05	0.13
Smoking^b^										
Never	6519 (42.0)	5734 (96.3)	1	1	1	1	1	1	1	1
Quit	2297 (14.8)	52 (0.9)	0.98 (−0.02)	0.81 (−0.21)	1.19 (0.18)”	1.12 (0.12)	1.15 (0.14)’	0.52 (− 0.66)	0.98 (− 0.02)	0.66 (− 0.41)
Current	6700 (43.2)	170(2.9)	1.06 (0.05)	1.30 (0.26)	1.53 (0.42)”	1.18 (0.17)	1.64 (0.50)”	1.72 (0.54)’	1.00 (0.00)	1.08 (0.08)
*P* for trend			0.11	0.19	< 0.001	0.35	< 0.001	< 0.05	0.91	0.91
Physical activity level^b^										
Low	10,438 (67.3)	3977 (66.8)	1	1	1	1	1	1	1	1
Moderate	4096 (26.4)	1704 (28.6)	0.87 (−0.15)”	1.06 (0.04)	0.75 (−0.28)”	1.01 (0.01)	0.83 (− 0.19)”	0.95 (− 0.05)	0.96 (− 0.04)	0.96 (− 0.04)
High	982 (6.3)	275 (4.6)	0.81 (− 0.21)’	0.92 (− 0.08)	0.63 (− 0.47)”	0.86 (− 0.15)	0.67 (− 0.40)”	0.79 (− 0.24)	0.98 (− 0.02)	0.84 (− 0.17)
*P* for trend			< 0.001	0.82	< 0.001	0.61	< 0.001	0.09	0.50	0.26

Continuous variables are mean ± SD, categorical variables are quantities with percentages in parentheses

‘*P* <  0.05. “*P* < 0.001. The odds ratios are calculated by logistic regression, significance is defined as *P* < 0.05

^a^ Adjusted for age (continuous); ^b^ Adjusted for age (continuous), BMI (continuous), WC (continuous), ethnicity and education

*OR* Odds ratio, *TC* Total cholesterol, *TG* Triglyceride, *HDL-C* High-density lipoprotein cholesterol, *LDL-C* Low-density lipoprotein cholesterol, *BMI* Body mass index, *WC* Waist circumstance

In the present study, five dietary patterns were extracted. The factor scores and names of the five dietary patterns are exhibited in Table 2. Factor 1 (meat) was largely consisted of meat, eggs, and fish, as well as bean products and alcohol. Factor 2 (high-energy) was loaded with high energy food, including sugary drinks, pickled food, fried food, and sweets. Factor 3 (high-protein) included coarse cereals, eggs, dairy, bean products and soybean milk. Factor 4 (healthy) was characterized by grain, coarse cereals, vegetables, and fruits, which are generally considered to be beneficial for health, while contained less alcohol and salt. Factor 5 (traditional Chinese) mainly includes grain, meat, pickled food, fried food, alcohol, salt, with less dairy and fruits. The five factors explained 49.6% of the variance in dietary intake, with patterns of “meat,” “high-energy,” “high-protein,” “healthy” and “traditional Chinese” accounting for 13.1%, 12.9%, 9.6%, 7.6%, 6.3%, respectively.

**Table 2:** Factor scores of five dietary patterns

	Factor 1 “meat”		Factor 2 “high-energy”		Factor 3 “high-protein”		Factor 4 “healthy”		Factor 5 “traditional Chinese”	
	Food	Factor score	Food	factor score	Food	factor score	Food	factor score	Food	factor score
Absolute value ≥ 0.2	Meat^a^	0.676	Sugary drinks	0.688	Coarse cereals^c^	0.519	Grain	0.399	Grain	0.588
	Eggs	0.457	Pickled food	0.229	Eggs	0.335	Coarse cereals	0.254	Meat	0.26
	Bean products^b^	0.231	Fried food	0.576	Dairy	0.505	Vegetables	0.762	Dairy	− 0.301
	Fish	0.731	Sweets	0.765	Bean products	0.555	Fruits	0.705	Fruits	−0.226
	Alcohol	0.574			Soybean milk	0.748	Alcohol	−0.302	Pickled food	0.611
							Salt	−0.223	Fried food	0.354
									Alcohol	0.281
									Salt	0.591
Absolute value < 0.2	Grain	0.035	Grain	−0.180	Grain	0.079	Meat	0.049	Coarse cereals	0.007
	Coarse cereals	0.048	Coarse cereals	−0.124	Meat	−0.033	Eggs	0.085	Eggs	−0.028
	Dairy	0.061	Meat	0.075	Vegetables	0.098	Dairy	0.068	Vegetables	0.045
	Vegetables	0.088	Eggs	0.049	Fruits	0.059	Bean products	0.079	Bean products	0.128
	Fruits	0.016	Dairy	0.104	Fish	0.136	Soybean milk	−0.131	Soybean milk	−0.019
	Soybean milk	−0.079	Vegetables	−0.161	Sugary drinks	−0.098	Fish	0.136	Fish	−0.134
	Sugary drinks	0.164	Fruits	0.175	Pickled food	−0.043	Sugary drinks	−0.084	Sugary drinks	0.045
	Pickled food	0.068	Bean products	−0.079	Fried food	0.124	Pickled food	−0.066	Sweets	−0.026
	Fried food	0.032	Soybean milk	0.055	Sweets	−0.010	Fried food	−0.121		
	Sweets	−0.115	Fish	0.069	Alcohol	−0.048	Sweets	0.133		
	Salt	0.114	Alcohol	− 0.178	Salt	−0.073				
			Salt	0.099						

^a^. Including poultry, pork, beef, mutton and so on

^b^. Processed food with soybeans, small beans, green beans, peas, broad beans and other beans as the main raw materials, including tofu and so on, but not soybean milk

^c^. Including sorghum, millet, buckwheat, oats and so on

The ORs of dyslipidemia according to quartiles of each pattern and PA level are presented in Table 3. After adjusted for age, BMI, WC, ethnicity, education, smoking and alcohol drinking status, quartile 3 and quartile 4 of the “meat” pattern were related to elevated risks of having a excess TC level (Q3: OR, 1.20; 95% CI, 1.10–1.32; Q4: OR, 1.42; 95% CI, 1.29–1.56), high LDL-C (Q3: OR, 1.18; 95% CI, 1.08–1.37; Q4: OR, 1.26; 95% CI, 1.16–1.37) and reduced risks of having a low HDL-C level(Q3: OR, 0.90; 95% CI, 0.82–0.99; Q4: OR, 0.77; 95% CI, 0.70–0.85) compared with quartile 1. Quartiles 3 and 4 of the “high-energy” pattern were related to elevated risk of excess LDL-C (Q3: OR, 1.09; 95% CI, 1.01–1.19; Q4: OR, 1.13; 95% CI, 1.04–1.23). Higher scores in the “high-protein” pattern showed a correlation with a decreased risk of abnormalities in each lipid index. The influence of “healthy” pattern on lipid profile was reflected in the decreased risk of high TC (Q4: OR, 0.87; 95% CI, 0.80–0.94) and high TG (Q3: OR, 0.88; 95% CI, 0.81–0.96; Q4: OR, 0.86; 95% CI, 0.79–0.94). The increased risk of high TG was found in quartiles 3 and 4 (Q3: OR, 1.10; 95% CI, 1.00–1.19; Q4: OR, 1.12; 95% CI, 1.03–1.23) of “traditional Chinese” pattern. Meanwhile, the individuals with moderate and high PALs were both at lower risk of abnormal TC (moderate: OR, 0.93; 95% CI, 0.87–0.99; high: OR, 0.84; 95% CI, 0.74–0.95), TG (moderate: OR, 0.83; 95% CI, 0.78–0.89; high: OR, 0.68; 95% CI, 0.60–0.78) and HDL-C (moderate: OR, 0.88; 95% CI, 0.82–0.94; high: OR, 0.73; 95% CI, 0.64–0.84). After the separate analysis of men and women, the associations were still significant among men while the effect of “meat” pattern on HDL-C and the associations between “high-protein,” “healthy” and “traditional Chinese” patterns with lipid profile were not significant in women. The relationship between moderate and high PALs with the decreased risk of hyperlipidemia was also only observed in men (Table 4).

**Table 3:** The ORs for abnormal levels of lipid-related indices according to categories of dietary patterns and physical activity level

	High TC		High TG		Low HDL-C		High LDL-C	
	OR (95% CI)	β	OR (95% CI)	β	OR (95% CI)	β	OR (95% CI)	β
Factor 1 “meat”								
Q1	1		1		1		1	
Q2	0.98 (0.89–1.06)	− 0.03	0.97 (0.89–1.06)	− 0.03	1.01 (0.92–1.10)	0.01	1.04 (0.95–1.12)	0.03
Q3	1.20 (1.10–1.32)”	0.19	0.97 (0.88–1.07)	−0.03	0.90 (0.82–0.99)’	− 0.10	1.18 (1.08–1.37)”	0.16
Q4	1.42 (1.29–1.56)”	0.35	1.05 (0.95–1.15)	0.04	0.77 (0.70–0.85)”	− 0.26	1.26 (1.16–1.37)”	0.23
Factor 2 “high-energy”								
Q1	1		1		1		1	
Q2	1.04 (0.96–1.12)	0.04	0.93 (0.86–1.01)	−0.07	0.94 (0.86–1.02)	− 0.07	1.07 (0.99–1.15)	0.06
Q3	1.00 (0.92–1.08)	0.00	0.87 (0.80–0.94)’	− 0.14	0.97 (0.89–1.05)	−0.03	1.09 (1.01–1.19)’	0.09
Q4	1.03 (0.95–1.12)	0.03	0.83 (0.76–0.90)”	−0.19	1.00 (0.92–0.10)	0.00	1.13 (1.04–1.23)”	0.12
Factor 3 “high-protein”								
Q1	1		1		1		1	
Q2	0.94 (0.86–1.02)	−0.07	0.85 (0.79–0.93)”	− 0.16	0.89 (0.82–0.97)”	−0.12	0.97 (0.89–1.05)	− 0.04
Q3	0.88 (0.81–0.96)’	− 0.13	0.86 (0.79–0.93)”	−0.15	0.91 (0.84–1.00)’	−0.09	0.91 (0.84–0.99)’	−0.09
Q4	0.83 (0.76–0.90)”	−0.19	0.80 (0.74–0.87)”	−0.22	0.92 (0.84–1.00)’	−0.09	0.86 (0.79–0.93)”	− 0.15
Factor 4 “healthy”								
Q1	1		1		1		1	
Q2	0.91 (0.84–0.99)’	− 0.09	0.96 (0.89–1.04)	− 0.04	1.11 (1.02–1.21)’	0.10	0.96 (0.88–1.04)	−0.05
Q3	0.94 (0.87–1.02)	− 0.06	0.88 (0.81–0.96)’	− 0.12	1.09 (1.00–1.19)	0.08	0.99 (0.91–1.07)	− 0.01
Q4	0.97 (0.80–0.94)’	− 0.14	0.86 (0.79–0.94)’	− 0.15	1.09 (1.00–1.19)	0.08	0.93 (0.86–1.01)	−0.07
Factor 5 “traditional Chinese”								
Q1	1		1		1		1	
Q2	0.96 (0.89–1.05)	− 0.04	1.02 (0.94–1.11)	0.02	1.01 (0.92–1.10)	0.01	1.01 (0.93–1.09)	0.01
Q3	1.04 (0.96–1.13)	0.04	1.10 (1.00–1.19)’	0.09	1.06 (0.97–1.16)	0.06	1.05 (0.97–1.14)	0.05
Q4	1.02 (0.93–1.11)	0.02	1.12 (1.03–1.23)’	0.12	1.04 (0.95–1.13)	0.04	0.97 (0.89–1.05)	−0.03
Physical activity level								
Low	1		1		1		1	
Moderate	0.93 (0.87–0.99)’	−0.08	0.83 (0.78–0.89)”	− 0.18	0.88 (0.82–0.95)”	− 0.13	0.97 (0.90–1.03)	− 0.04
High	0.84 (0.74–0.95)’	− 0.18	0.68 (0.60–0.78)”	−0.39	0.73 (0.64–0.84)”	− 0.31	0.95 (0.84–1.08)	−0.05

Adjusted for age (continuous), BMI (continuous), WC (continuous), sex, ethnicity, education, alcohol drinking status and smoking status

‘*P* < 0.05. “*P* < 0.001. The odds ratios were calculated by logistic regression, significance is defined as *P* < 0.05

*OR* Odds ratio, *TC* Total cholesterol, *TG* Triglyceride, *HDL-C* High-density lipoprotein cholesterol, *LDL-C* Low-density lipoprotein cholesterol

**Table 4:** The ORs for abnormal levels of lipid-related indices separated by sex

	High TC		Female		High TG		Female	
	Male				Male			
	OR (95% CI)	β	OR (95% CI)	β	OR (95% CI)	β	OR (95% CI)	β
Factor 1 “meat”								
Q1	1		1		1		1	
Q2	1.06 (0.96–1.17)	0.06	1.01 (0.86–1.19)	0.01	0.95 (0.87–1.05)	− 0.05	0.83 (0.69–1.00)	− 0.18
Q3	1.31 (1.19–1.46)”	0.27	1.04 (0.89–1.23)	0.04	0.99 (0.90–1.10)	− 0.01	0.89 (0.74–1.07)	− 0.12
Q4	1.48 (1.33–1.64)”	0.39	1.18 (1.00–1.40)’	0.17	1.07 (0.97–1.19)	0.07	0.74 (0.62–0.90)’	−0.30
Factor 2 “high-energy”								
Q1	1		1		1		1	
Q2	1.05 (0.95–1.15)	0.05	1.11 (0.94–1.29)	0.10	0.91 (0.83–1.00)’	− 0.10	0.94 (0.78–1.12)	− 0.06
Q3	1.01 (0.92–1.11)	0.01	1.03 (0.88–1.22)	0.03	0.87 (0.79–0.95)’	− 0.14	0.92 (0.76–1.11)	−0.08
Q4	1.07 (0.97–1.17)	0.06	1.04 (0.88–1.22)	0.04	0.86 (0.79–0.95)’	− 0.15	0.81 (0.66–0.98)’	− 0.22
Factor 3 “high-protein”								
Q1	1		1		1		1	
Q2	0.91 (0.83–1.00)’	−0.10	1.01 (0.86–1.18)	0.01	0.83 (0.76–0.91)”	−0.19	0.94 (0.78–1.14)	− 0.06
Q3	0.89 (0.81–0.98)’	− 0.11	0.93 (0.79–1.09)	− 0.08	0.85 (0.77–0.93)’	− 0.17	0.88 (0.73–1.06)	− 0.13
Q4	0.80 (0.73–0.88)”	−0.22	0.98 (0.83–1.15)	− 0.02	0.80 (0.73–0.88)”	− 0.22	0.85 (0.71–1.03)	− 0.16
Factor 4 “healthy”								
Q1	1		1		1		1	
Q2	0.90 (0.81–0.98)’	− 0.11	0.92 (0.78–1.08)	− 0.08	0.97 (0.88–1.06)	− 0.03	0.97 (0.80–1.17)	− 0.03
Q3	0.97 (0.88–1.06)	−0.03	0.94 (0.80–1.10)	− 0.06	0.88 (0.81–0.97)’	− 0.12	0.92 (0.76–1.11)	−0.09
Q4	0.85 (0.77–0.93)’	− 0.17	0.86 (0.73–1.01)	− 0.16	0.85 (0.78–0.94)’	− 0.16	0.92 (0.76–1.12)	− 0.08
Factor 5 “traditional Chinese”								
Q1	1		1		1		1	
Q2	1.09 (0.99–1.20)	0.09	0.99 (0.85–1.17)	−0.01	1.12 (1.02–1.23)’	0.11	1.07 (0.88–1.29)	0.07
Q3	1.10 (1.00–1.21)	0.09	0.96 (0.82–1.13)	−0.04	1.10 (1.00–1.21)’	0.10	0.98 (0.81–1.19)	− 0.02
Q4	1.10 (1.00–1.21)	0.10	0.88 (0.75–1.03)	−0.13	1.21 (1.10–1.33)”	0.19	0.96 (0.79–1.16)	− 0.04
	Low HDL-C				High LDL-C			
	Male		Female		Male		Female	
	OR (95% CI)	β	OR (95% CI)	β	OR (95% CI)	β	OR (95% CI)	β
Factor 1 “meat”								
Q1	1		1		1		1	
Q2	0.95 (0.86–1.05)	−0.05	0.97 (0.83–1.14)	− 0.03	1.09 (0.99–1.19)	0.08	0.92 (0.79–1.08)	−0.08
Q3	0.80 (0.72–0.89)”	− 0.23	0.96 (0.82–1.14)	−0.04	1.20 (1.09–1.32)”	0.18	0.98 (0.84–1.15)	− 0.02
Q4	0.73 (0.65–0.81)”	−0.32	0.96 (0.81–1.13)	− 0.05	1.24 (1.13–1.36)”	0.22	1.19 (1.01–1.39)’	0.17
Factor 2 “high-energy”								
Q1	1		1		1		1	
Q2	0.97 (0.88–1.07)	−0.03	0.89 (0.76–1.05)	− 0.12	1.05 (0.96–1.16)	0.05	1.11 (0.95–1.29)	0.10
Q3	1.03 (0.93–1.14)	0.03	0.86 (0.73–1.02)	−0.15	1.14 (1.04–1.25)’	0.13	0.99 (0.84–1.16)	−0.01
Q4	1.04 (0.94–1.15)	0.04	0.93 (0.79–1.09)	−0.08	1.17 (1.02–1.28)’	0.15	1.09 (0.93–1.28)	0.08
Factor 3 “high-protein”								
Q1	1		1		1		1	
Q2	0.89 (0.80–0.98)’	−0.12	0.88 (0.75–1.04)	− 0.12	0.95 (0.87–1.04)	−0.05	1.02 (0.87–1.20)	0.02
Q3	0.91 (0.83–1.01)	−0.09	0.91 (0.77–1.07)	−0.10	0.93 (0.85–1.02)	− 0.07	0.93 (0.79–1.09)	−0.08
Q4	0.94 (0.85–1.03)	−0.07	0.89 (0.75–1.04)	− 0.12	0.89 (0.81–0.98)’	− 0.11	0.86 (0.74–1.02)	− 0.15
Factor 4 “healthy”								
Q1	1		1		1		1	
Q2	1.07 (0.97–1.19)	0.07	0.97 (0.82–1.14)	−0.03	0.96 (0.88–1.06)	− 0.04	0.89 (0.76–1.04)	− 0.12
Q3	1.09 (0.98–1.21)	0.09	1.00 (0.85–1.17)	0.00	1.04 (0.95–1.14)	0.04	0.90 (0.77–1.06)	−0.10
Q4	1.08 (0.98–1.20)	0.08	1.09 (0.92–1.28)	0.09	0.91 (0.83–1.00)’	− 0.10	0.89 (0.76–1.05)	− 0.11
Factor 5 “traditional Chinese”								
Q1	1		1		1		1	
Q2	1.03 (0.93–1.14)	0.03	0.99 (0.84–1.17)	−0.01	1.10 (1.00–1.20)	0.09	1.04 (0.89–1.23)	0.04
Q3	1.00 (0.90–1.11)	0.00	0.97 (0.83–1.15)	−0.03	1.05 (0.96–1.16)	0.05	0.98 (0.84–1.15)	−0.02
Q4	1.05 (0.95–1.17)	0.05	1.03 (0.88–1.22)	0.03	1.00 (0.91–1.09)	−0.01	1.01 (0.86–1.19)	0.01

Adjusted for age (continuous), BMI (continuous), WC (continuous), ethnicity, education, alcohol drinking status and smoking status

‘*P* < 0.05. “*P* < 0.001. The odds ratios were calculated by logistic regression, significance is defined as *P* < 0.05

*OR* Odds ratio, *TC* Total cholesterol, *TG* Triglyceride, *HDL-C* High-density lipoprotein cholesterol, *LDL-C* Low-density lipoprotein cholesterol

The interactions between PA level and diet patterns evaluated by stratified analyses are exhibited in Table 5. The dietary patterns “high-energy,” “healthy” and “traditional Chinese” were found to interact significantly with PA in their effect of TG. Similar interactions were also observed between PA with patterns “meat” and “traditional Chinese” on TG and pattern “healthy” on LDL-C. The results also indicated that participants had high level of PA with lower factor scores in “meat” pattern and moderate factor scores for “healthy” and “traditional Chinese” pattern showed the lowest risk for high TC (meat: OR, 0.67; 95% CI, 0.51–0.86; healthy: OR, 0.67; 95% CI, 0.53–0.83; traditional Chinese: OR, 0.62; 95% CI, 0.48–0.80). The lowest risk of high TG was found in physically active subjects with medium factor scores in “high-energy” pattern (OR, 0.54; 95% CI, 0.43–0.68) and high factor scores for “healthy” pattern (OR, 0.58; 95% CI, 0.47–0.72). Individuals with high level of PA and certain factor scores were also found to be associated with the lowest risk of low HDL-C (high in “meat” pattern: OR, 0.58; 95% CI, 0.46.0.73; medium in “high-energy” pattern: OR, 0.53; 95% CI, 0.41–0.68) and high LDL-C (high in “high-protein”: OR, 0.78; 95% CI, 0.65–0.94; low in “healthy”: OR, 0.74; 95% CI, 0.57–0.95).

**Table 5:** Interaction between dietary patterns and physical activity level on the risk of abnormal lipid-related indices

		High TC		High TG		Low HDL-C		High LDL-C	
		OR (95% CI)	β	OR (95% CI)	β	OR (95% CI)	β	OR (95% CI)	β
Factor 1 “meat”									
Low	PAL low	1		1		1		1	
	PAL moderate	0.96 (0.86–1.08)	−0.04	0.95 (0.84–1.06)	− 0.06	0.92 (0.82–1.03)	− 0.08	0.90 (0.81–1.01)	− 0.10
	PAL high	0.67 (0.51–0.86)’	− 0.41	0.65 (0.50–0.84)’	− 0.44	0.65 (0.51–0.84)’	− 0.43	0.80 (0.63–1.01)	− 0.22
Medium	PAL low	1.00 (0.92–1.10)	0.00	0.95 (0.87–1.05)	−0.05	0.92 (0.84–1.01)	− 0.08	1.00 (0.92–1.09)	0.00
	PAL moderate	1.00 (0.89–1.13)	0.00	0.85 (0.76–0.96)’	− 0.16	0.83 (0.73–0.93)’	− 0.19	1.02 (0.91–1.14)	0.02
	PAL high	0.99 (0.80–1.23)	−0.01	0.83 (0.66–1.03)	−0.19	0.69 (0.55–0.87)’	−0.37	1.14 (0.92–1.40)	0.13
High	PAL low	1.39 (1.27–1.53)”	0.33	1.15 (1.04–1.27)’	0.14	0.78 (0.70–0.86)”	− 0.25	1.18 (1.09–1.29)”	0.17
	PAL moderate	1.15 (1.02–1.31)’	0.14	0.82 (0.72–0.93)’	− 0.20	0.59 (0.52–0.68)”	−0.52	1.14 (1.01–1.28)’	0.13
	PAL high	1.09 (0.89–1.34)	0.09	0.69 (0.56–0.85)”	− 0.38	0.58 (0.46–0.73)”	− 0.55	1.03 (0.85–1.26)	0.03
*P* for interaction		0.41		0.01		0.39		0.47	
Factor 2 “high-energy”									
Low	PAL low	1		1		1		1	
	PAL moderate	0.89 (0.80–1.00)’	− 0.12	0.85 (0.76–0.95)’	−0.16	0.84 (0.75–0.95)’	− 0.17	0.97 (0.87–1.08)	− 0.03
	PAL high	0.76 (0.62–0.93)’	− 0.28	0.70 (0.57–0.86)’	− 0.36	0.84 (0.68–1.04)	− 0.17	0.92 (0.76–1.12)	− 0.09
Medium	PAL low	1.00 (0.91–1.09)	− 0.01	0.90 (0.82–0.98)’	− 0.11	1.05 (0.96–1.15)	0.05	1.07 (0.98–1.16)	0.06
	PAL moderate	0.88 (0.78–0.99)’	− 0.13	0.73 (0.65–0.82)”	−0.32	0.85 (0.75–0.96)’	− 0.17	0.98 (0.88–1.10)	− 0.02
	PAL high	0.79 (0.63–1.00)’	− 0.23	0.54 (0.43–0.68)”	− 0.62	0.53 (0.41–0.68)”	− 0.64	0.93 (0.75–1.15)	− 0.08
High	PAL low	0.98 (0.90–1.07)	− 0.02	0.83 (0.76–0.91)”	− 0.18	0.98 (0.90–1.07)	− 0.02	1.10 (1.01–1.20)’	0.09
	PAL moderate	1.01 (0.89–1.14)	0.01	0.70 (0.61–0.79)”	−0.36	0.96 (0.84–1.09)	− 0.05	1.11 (0.99–1.26)	0.11
	PAL high	0.95 (0.75–1.21)	− 0.05	0.65 (0.51–0.84)’	− 0.43	0.77 (0.60–0.99)’	− 0.27	1.20 (0.96–1.50)	0.18
*P* for interaction		0.03		0.88		0.53		0.34	
Factor 3 “high-protein”									
Low	PAL low	1		1		1		1	
	PAL moderate	0.92 (0.81–1.04)	− 0.09	0.85 (0.75–0.97)’	− 0.16	0.80 (0.70–0.91)’	− 0.23	0.94 (0.84–1.07)	− 0.06
	PAL high	0.90 (0.70–1.17)	−0.10	0.64 (0.49–0.84)’	− 0.44	0.68 (0.51–0.90)’	− 0.38	1.10 (0.86–1.41)	0.10
Medium	PAL low	0.96 (0.88–1.04)	− 0.04	0.90 (0.83–0.98)’	− 0.11	0.91 (0.84–1.00)’	− 0.09	1.00 (0.92–1.09)	0.00
	PAL moderate	0.87 (0.78–0.98)’	− 0.14	0.79 (0.70–0.89)”	− 0.24	0.86 (0.77–0.97)’	− 0.15	0.95 (0.85–1.06)	− 0.05
	PAL high	0.77 (0.62–0.96)’	− 0.26	0.62 (0.49–0.77)”	− 0.49	0.68 (0.53–0.86)’	− 0.39	0.95 (0.77–1.17)	− 0.05
High	PAL low	0.84 (0.77–0.92)”	− 0.18	0.90 (0.82–0.98)’	− 0.10	0.96 (0.87–1.05)	− 0.05	0.87 (0.80–0.95)’	− 0.14
	PAL moderate	0.82 (0.74–0.92)”	−0.19	0.75 (0.67–0.83)”	− 0.29	0.83 (0.74–0.92)’	− 0.19	0.88 (0.80–0.98)’	− 0.12
	PAL high	0.71 (0.59–0.87)’	− 0.34	0.69 (0.56–0.84)”	− 0.38	0.68 (0.55–0.83)”	− 0.39	0.78 (0.65–0.94)’	− 0.25
*P* for interaction		0.84		0.55		0.49		0.60	
Factor 4 “healthy”									
Low	PAL low	1		1		1		1	
	PAL moderate	0.88 (0.77–0.99)’	− 0.13	0.81 (0.72–0.92)’	− 0.21	0.82 (0.72–0.94)’	− 0.20	0.92 (0.81–1.03)	− 0.09
	PAL high	0.72 (0.56–0.93)’	− 0.33	0.63 (0.49–0.82)”	− 0.46	0.66 (0.50–0.88)’	− 0.42	0.74 (0.57–0.95)’	− 0.31
Medium	PAL low	0.92 (0.85–1.00)’	− 0.08	0.90 (0.83–0.98)’	− 0.11	1.04 (0.95–1.14)	0.04	0.98 (0.90–1.06)	− 0.02
	PAL moderate	0.87 (0.78–0.98)’	− 0.14	0.77 (0.68–0.86)”	− 0.27	0.85 (0.75–0.95)’	− 0.17	0.93 (0.84–1.04)	− 0.07
	PAL high	0.67 (0.53–0.83)”	− 0.41	0.75 (0.60–0.93)’	− 0.29	0.75 (0.60–0.95)’	− 0.29	0.87 (0.71–1.07)	− 0.14
High	PAL low	0.86 (0.78–0.94)’	− 0.15	0.88 (0.80–0.96)’	− 0.13	1.05 (0.96–1.15)	0.05	0.89 (0.81–0.97)’	− 0.12
	PAL moderate	0.84 (0.75–0.93)’	− 0.18	0.77 (0.69–0.86)”	−0.26	0.99 (0.89–1.11)	− 0.01	0.91 (0.82–1.01)	−0.10
	PAL high	0.88 (0.73–1.07)	−0.12	0.58 (0.47–0.72)”	−0.54	0.75 (0.61–0.93)’	−0.29	1.03 (0.85–1.23)	1.03
*P* for interaction		0.01		0.44		0.18		0.01	
Factor 5 “traditional Chinese”									
Low	PAL low	1		1		1		1	
	PAL moderate	0.96 (0.86–1.08)	−0.04	0.96 (0.85–1.08)	−0.05	0.85 (0.76–0.96)’	− 0.16	0.95 (0.85–1.06)	−0.05
	PAL high	1.14 (0.94–1.39)	0.13	0.83 (0.67–1.02)	−0.19	0.69 (0.55–0.85)’	− 0.38	1.11 (0.92–1.34)	0.10
Medium	PAL low	1.08 (0.98–1.17)	0.07	1.13 (1.03–1.24)’	0.12	1.00 (0.91–1.09)	0.00	1.06 (0.97–1.16)	0.06
	PAL moderate	0.97 (0.86–1.09)	−0.03	0.89 (0.79–1.01)	− 0.11	0.85 (0.75–0.96)’	− 0.17	1.00 (0.89–1.12)	0.00
	PAL high	0.62 (0.48–0.80)”	−0.48	0.71 (0.56–0.90)’	−0.34	0.70 (0.54–0.89)’	−0.36	0.83 (0.66–1.04)	− 0.19
High	PAL low	1.05 (0.96–1.15)	0.05	1.18 (1.08–1.30)”	0.17	0.98 (0.90–1.08)	−0.02	0.99 (0.91–1.08)	−0.01
	PAL moderate	0.97 (0.86–1.09)	−0.03	0.95 (0.84–1.08)	−0.05	0.89 (0.79–1.01)	− 0.11	0.99 (0.88–1.11)	−0.01
	PAL high	0.78 (0.62–0.99)’	−0.25	0.77 (0.61–0.98)’	− 0.26	0.74 (0.57–0.95)’	− 0.31	0.91 (0.73–1.14)	−0.10
*P* for interaction		0.02		0.02		0.38		0.58	

Dietary patterns were classified according to the tertiles of factor scores

Adjusted for age (continuous), BMI (continuous), WC (continuous), ethnicity, education, alcohol drinking status and smoking status

‘*P* < 0.05. “*P* < 0.001. The ORs (95% CI) were calculated by logistic regression, significance is defined as *P* < 0.05

*OR* Odds ratio, *CI* Confidence interval, *TC* Total cholesterol, *TG* Triglyceride, *HDL-C* High-density lipoprotein cholesterol, *LDL-C* Low-density lipoprotein cholesterol, *PAL* Physical activity level

## Discussion

The present study demonstrated the relationship between dietary pattern, PAL, and lipid-related indices in a sizeable Chinese population. Higher factor scores of “high-protein” pattern and “healthy” pattern were related to lower risk of abnormal lipid indices. Higher PALs were also associated with decreased risk of TC, TG and HDL-C abnormalities. In addition, lifestyle factors were more significantly related to blood lipids in men than women.

High factor scores with a “high-protein” pattern, which consisted of high-quality protein foods, were found related to lower risk of lipid abnormalities. The foods loaded in this pattern are commonly considered to be beneficial for health [23]. Dietary fiber from coarse cereals in “high-protein” mode is also considered to be beneficial to lipid profiles [24]. The diet pattern that mainly consumes meat is considered to be harmful to blood lipid, while quartiles 3 and 4 of “meat” pattern in the present study were observed to be related to decreased risk of low HDL-C. This may because the high factor load of alcohol in the “meat” pattern of this study, which is considered a light to moderate consumption could elevate HDL-C concentration to achieve a cardio-protective effect [25, 26]. The high consumption of fish in “meat” pattern could be another potential influence factor to improve HDL-C by the high content of n-3 PUFA [27]. Studies suggested that diet pattern with more fruits and vegetables is beneficial to health problems including adiposity, metabolic syndrome, and cancer [28–30]. The associations between “healthy” pattern and lower risk of abnormal TC and TG in this study is consistent with these studies.

Previous studies suggested that diets with high energy food such as saturated fat, sodium and sugar are related to dyslipidemia [10, 11]. On the contrary, the higher factor score of “high-energy” pattern in this study were found to be related to decreased risk of excess TG. This may be due to the “high-energy” pattern in this study was mainly contributed by sugary drinks, fried foods, and sweets. This kind of diet mode is more achieved by young people. They usually pay more attention to PA and consume less carbohydrate and more high-quality protein foods [31]. Studies have shown that increasing protein intake at the expense of carbohydrate may decreased cardiometabolic risk [32] because consume more protein instead of carbohydrate seems could improve lipid profiles [33–35].

In consist with previous studies, individuals with moderate and high PALs were at lower risk of TC, TG, and HDL-C abnormalities than those who were inactive. The study of Gibbs et al. provided the evidence that all kind of PA are related to lower risk of cardiovascular disease including abnormal lipid profile, regardless of cardiometabolic risk [36]. However, the effect of exercise on LDL-C was not significant in this study. This may because that changes in LDL-C is not necessarily be expected in response to exercise training. Benefits of exercise on serum lipids are primarily though effects on TG, sometimes with a modest effect on HDL-C [37].

Gender has been proven important in the relationship between lifestyle factors and lipids. The differences in blood lipid concentration between male and female can be attributed to various factors including estrogens, serum adiponectin and different social life [38–42]. Estrogens have been reported to inhibit HDL-C catabolism by reducing hepatic lipase activity and to increase LDL-C catabolism by elevate the amount of LDL-C receptor [38–40]. A study revealed a higher concentration of serum adiponectin, which is closely associated with favorable lipid profile, in women than in men [41]. As for the difference in PA, it may because that Chinese women’s PA mainly focuses on housework, which is not well reflected in the evaluation method used on PAL. However, more household PA is found to be related to the improvement of blood lipid [42]. Research evidence shows that there are also gender differences in the effect of exercise response, particularly the acute exercise response, on blood lipid metabolism [43]. Therefore, further studies are needed to explore the relationship between more comprehensive evaluation of PAL and dyslipidemia.

Through the interaction analysis, dietary patterns and PA were found to be interact with each other in terms of their impact on certain indices. The “high-protein” pattern and “healthy” pattern, which were closely associated with favorable lipid profiles, did not show significant interactions with PA in the effect on the risk of dyslipidemia. Previous study suggested that healthy behaviors such as good eating pattern and exercise habits usually exist at the same time [44]. While the interactions were found in the patterns related to higher risk of dyslipidemia such as “meat,” “high-energy” and “traditional-Chinese”. This may indicate that higher PA can offset the negative effects of some unhealthy dietary structure on blood lipids. Thus, it is essential to maintain adequate PA and follow a diet with high consumption of high-quality protein, vegetables, and fruits to achieve healthy lipid profiles in the general population.

### Study strength and limitations

Strengths of the present study is the large sample size and the use of factor analysis, which has emerged as a practical way to deliver information to the public and to implement policies [45]. There are some limitations need to be considered in the present study. First, the data of PA and eating habits obtained by retrospective questionnaire might be influenced by recall bias. Face-to-face interviews were conducted by well trained and certified personnel to reduce potential information bias. Second, leisure time PA was not included in the PAL evaluation. Usually this type of PA is not accurately recorded in daily life, which is difficult to obtain through a questionnaire. Well-designed cohort studies are needed to explore the relationship between leisure time PA and lipid profile. Last, the effect of diet and PA on blood lipid may act through changes in bodyweight, adjusting BMI and WC as covariates could lead to a worsened ability to discover the effects. All the analysis was repeated without adjusting the two covariates, and the results remained unchanged.

## Conclusions

In conclusion, this study demonstrated the effect of dietary patterns and PA on lipid-related indices, as well as their interaction in Chinese population. PA and a dietary pattern with high consumption of high-quality protein, vegetables, and fruits are worthy of promotion in the general population to maintain healthy lipid profile and prevent the occurrence or progression of dyslipidemia and other chronic disease. In addition, gender is an important factor need to be considered in the management and intervention of dyslipidemia, since there are differences in the effect of lifestyle factors across genders.

## Data Availability

The data used in this study are confidential.
